# Supplementary material for: Optimized processing and analysis of conventional confocal microscopy generated scanning FCS data
Source: Methods. 2018 May 1;140-141:62–73. doi: 10.1016/j.ymeth.2017.09.010 (PMC6026296; doi:10.1016/j.ymeth.2017.09.010)
Supplement: Supplementary data 1 [file mmc1.pdf]

FoCuS-scan User Manual v1.2 (for FoCuS-scan v1.14.78+)

By Dr. Dominic Waithe

Introduction

Scanning Fluorescence Correlation Spectroscopy (scanning FCS) is a variant of the conventional point FCS and allows diffusion at multiple locations to be measured with high spatiotemporal synchronisation. In scanning FCS, the laser illumination spot is scanned repetitively across a sample in a line (or circle) and the light emitted from each point detected in sequence. The FoCuS-scan software provides an end-to-end solution for processing and analysing scanning data produced using commercial turn-key systems. As part of this package, the FoCuS-scan software contains tools that allow photobleaching artefacts to be compensated for, as well as tools that allow cropping to be applied to samples as well as a host of innovative visualisation techniques. Furthermore, the FoCuS-scan software utilises advanced fitting algorithms which accompany the data processing and allow large and complex datasets to be efficiently analysed. The following document is designed to be a detailed resource for the use of FoCuS-scan software. It does not represent an exhaustive guide to correlation and fitting of correlation functions but it does describe the tools and resources within the FoCuS-scan software.

Scanning FCS note on acquisition

For a scanning FCS measurement the fluorescence intensity of the sample across a laser line is systematically collected for the duration of the experiment The laser focus is moved over the specimen, recording at each of M positions, before repeating the cycle N times. The duration spent scanning across a pixel location (e.g. M=0) is known as the dwell time, whereas the time taken to repeat one of N cycles is denoted the line-time. On most commercial turnkey systems the laser can only be configured to scan along a line on the sample and so there is delay whilst the laser focus is moved back to the origin. With the correct equipment however, it is possible to scan a circle, in which case the integrated dwell time for all M locations and the line time will be the same. The pixel size is typically in the range of 80-150 nm and the length of the line typically around 5 um with often M=64 locations specified. Experiments can be varied in but between 10-60 s is a practical range depending on the speed of the species being studied. Data is then exported as an intensity carpet from the microscope in one of the accepted formats for the FoCuS-scan software (.msr, .lsm, .lif or OME-TIFF). The exported intensity carpets can be directly imported into the FoCuS-scan software, if the format is not compatible we recommend using software such as ImageJ/Fiji to convert the file into a OME-TIFF file before proceeding.

Instructions for using the scanning interface

Below are the instructions for using the FoCuS-scan software. The most important button to start with is the ‘Open File’ button marked just below position (5) in the below figure. Click ‘Open File’ and then select a file to proceed with the software. The second most important button is the ‘Export to Fit’ button shown to the left of (19). This button will export the correlated carpet into the FCS Equation Fitting tab, which is accessible through clicking the tab at the top of the software (8).

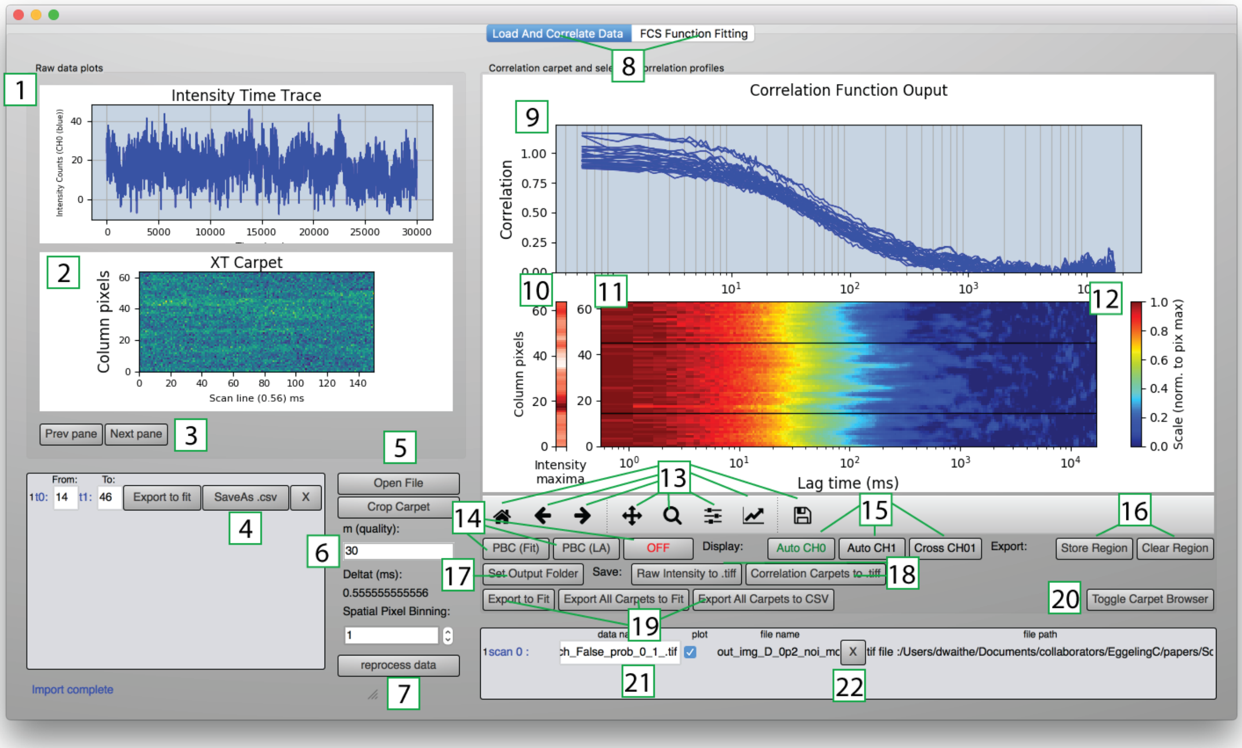

NB Whilst using the software, if you place the mouse cursor over a button a tooltip will appear explaining the function of a button.

1) Intensity Time Trace:
- This plot represents the integrated intensity of the carpet at each time-point.
Tip: If the average of this trace decreases over time it is likely that photobleaching is occurring during your acquisition. Consider applying photobleaching correction to your input data.

2) XT Carpet:
- The XT carpet visualisation shows the intensity levels of the raw carpet input. The intensity carpet is very long and so only a small section is visualised.

3) Through using the 'Prev pane' and 'Next' pane buttons it is possible to move through the intensity trace sections.

4) Stored Regions Manager:

- This table allows you to review stored regions selected on the Correlation Carpet Visualisation (11) and stored with ‘Store Region’ button (16).
Tip: This table allows you to make a note of the exact spatial ranges selected and also to export specific ranges from each carpet.

5) Import and pre-processing options
- Open Files: - Click this and use the dialog to select and open single or multiple files for correlation ((.msr, .lsm, .lif or OME-TIFF).
-Crop Carpet : - Applies a Crop to the software in either spatially or temporally

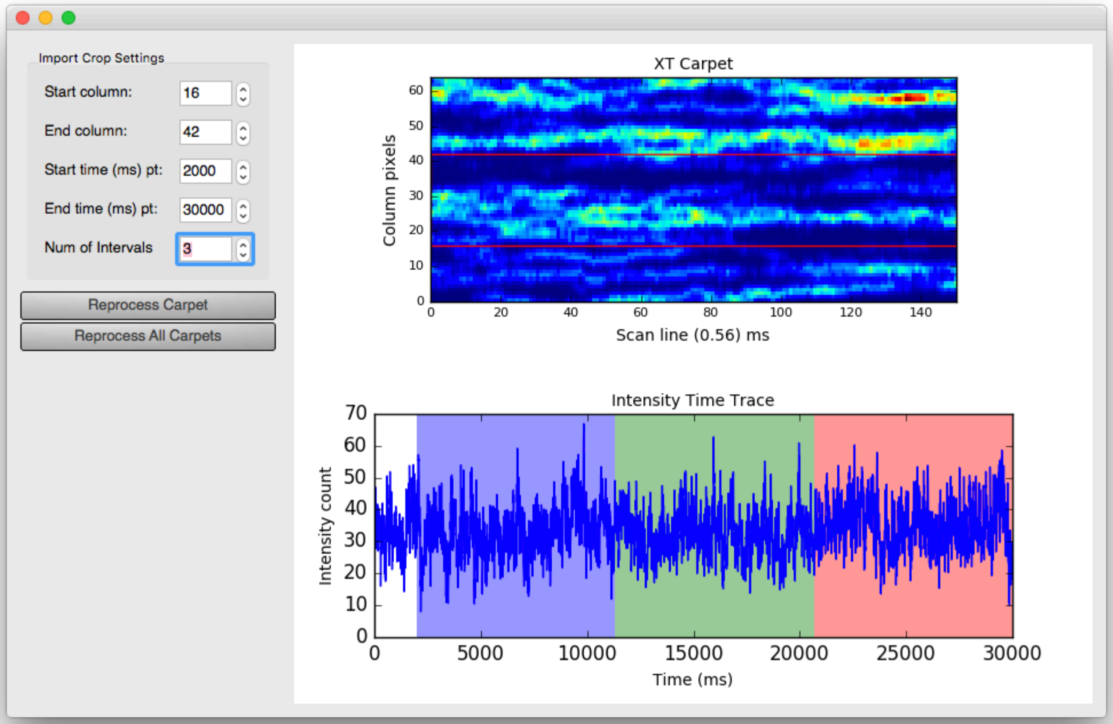

Within Plugin:

- ‘Start column’ select the pixel number to start the crop spatially.
- ‘End column’ select the pixel number to end the crop spatially.
- ‘Start time’ select the time-point to start the crop temporally.
- ‘End time’ select the time-point to end the crop temporally.
- Number of Intervals. If you wish you can split the cropped region into a number of equally sized regions.
- ‘Reprocess Carpet’ button will apply to the currently selected carpets
- ‘Reprocess All Carpets’ button will apply to all the loaded carpets
  Tip: Remember to check ‘plot’ located below ‘7’ for one of your data-files back in the main software interface to visualise your cropped data.

6) Correlation parameters:

-‘ m (quality): ’, this is represents the number of points to be calculated for each log-level of tau during correlation. Increasing this number will increase the resolution of the correlation.
- ‘Deltat (ms):', The calculated scanning line time in ms.

- ‘Spatial Pixel Binning: ', this represents the number of pixels to integrate spatially before correlation. Increasing the number will reduce noise at the cost of your spatial resolution. Although this can be effective for reducing the impact of noise, the likely impact of applying this technique will be that your measured transit times will increase as a result of the lengthened effective pixel size and the effect this has on the measurement PSF.

7) ‘Reprocess data’. Click this to apply changes made to the correlation parameters ‘m’ or ‘Spatial Pixel Binning' for all open data.

8) Click “FCS Function Fitting” to change the tab and reveal the fitting section.

9) Correlation Function Profile Plot:
- This plot will depict the correlation functions calculated from the spatial pixels selected in the Correlation Carpet Visualisation.
- *Tip: By clicking and drawing a range on this plot you can set the minima and maxima of the visualised correlation carpet.*

10) The corresponding maximum pixel intensity for each pixel of the correlation is shown here.

11) Correlation Carpet Visualisation:
- The colour of the carpet represents the correlation function output at each correlation lag time (horizontal) and at each scan-linepixel(vertical). To visualise as subset of pixels, click and left-click and drag on the carpet image.

12) The colorbar legend is located to the right of the plot and depicts the colour used for varying levels of correlation.

13) Below the carpet is a Toolbar. The tools on the toolbar allow manipulation of the correlation carpets. A) House: will return settings to default, click if you want to undo any changes you make to the visualisation. B and C) Arrows, will redo and undo changes in sequence. D) Multi-directional Arrows, when active, can be used to click and drag the visualisations. D) Zoom, when active allows the user to zoom in and out of the visualisations. E) Margins, allows the user to change the margins and padding for the visualisations (useful for creating figures). F) Disk, allows the user to save out an image of the correlation plots. G) Tick, Allows the user to format the visualisation of the plots and to specify ranges.

14) Photobleaching correction and correlation plot control buttons
PBC (Fit): - Photobleaching correlation using a mono-exponential fit as the correction.
Tip: To toggle the correction for comparison click the 'C1 ON' or the 'OFF' button.

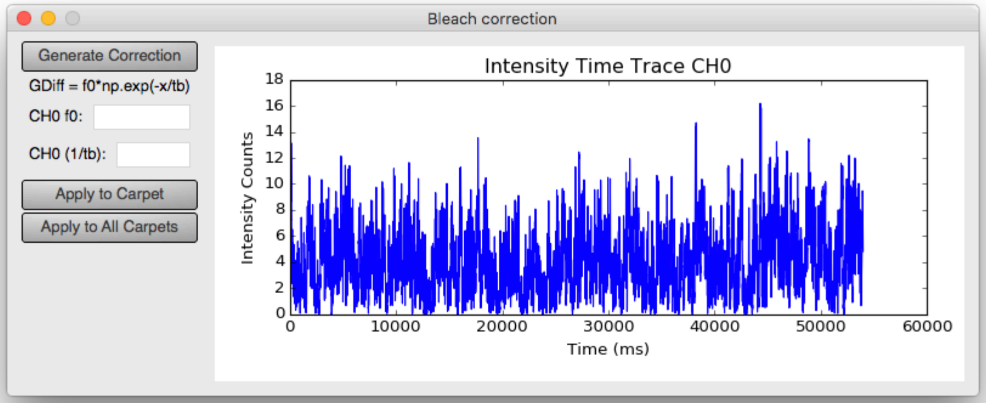

Within Plugin:

- Generate Correction: Calculates and visualises the correction.

Tip: The red-line represents the fitted equation. The better this fits the time-series data the better the output profile will be.

- GDiff = f0*np.exp(-x/tb) represents the function used to calculate the correction.
- Apply to selected carpet: Will apply the correction to the presently selected carpet.
- Apply to All Carpets: Will calculate and apply a correction for each carpet loaded in the data viewer.

PBC (LA): - Photobleaching correlation using local averaging. Clicking this will open the plugin for performing this correction.

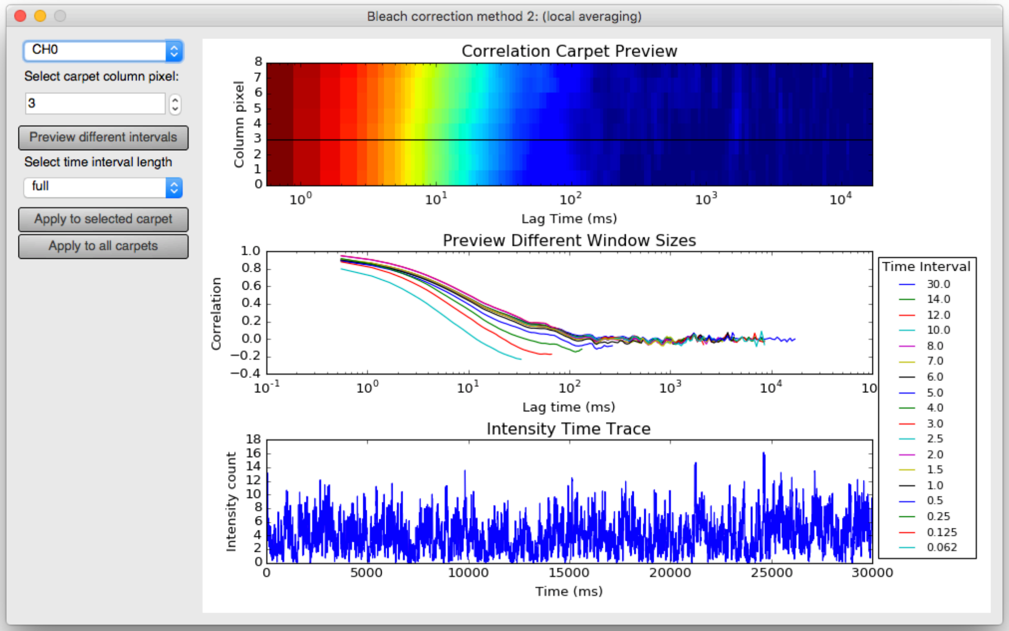

Tip: To toggle the correction for comparison click the 'C2 ON' or the 'OFF' button.

Within Plugin:

Plug-In: Bleach correction method 2: (local averaging).

- Select Channel. View either channel 0 or channel 1 (if available).
- Optional: Select a particular pixel on which to test the analysis.
- Optional: Preview different intervals. Will apply correction on a single selected pixel (selected above to allow preview of different time intervals).
- Selected Time Interval: This is the Time Interval which will be used for the local averaging correction.
- Apply to selected carpet: Will apply the local averaging to the presently selected carpet.
- Apply to all carpets: Will apply the local averaging to each carpet loaded in data viewer.

Tip: When you choose the Time Interval, you should choose a sufficiently small interval to remove photobleaching artefacts from resulting carpet, but as long an interval as possible to preserve the transit time.

Tip: The selected Time Interval will be visualised on the Intensity Time Trace as alternating colours.

15) Visualisation Buttons. 'Auto CH0' refers to the 1st channel auto-correlation, 'Auto CH1' refers to the 2nd channel auto-correlation (if present), 'Cross CH01' refers to the cross-correlation with respect to the 1st channel to the 2nd (if 2nd channel is present).

16) Selection Buttons. Store Region' button will add to the 'Stored Regions Manager' a selection drawn on the 'Correlation Carpet Visualization'.
-'Clear Region' will clear any region drawn on the 'Correlation Carpet Visualization', ensuring all pixels will be exported.

17) 'Set Output Folder', this button opens a dialog which will allow you to set a directory for the export of files using any of the 'Raw Carpet', 'Log Norm. Carpet' and 'Export All carpets to CSV' buttons.

18) Export .tiff files control buttons.
- 'Raw Intensity to .tiff' exports raw intensity data as a .tiff file. This is a useful step for converting image files from specific formats (e.g. lsm) into a tiff. It can also be used as mechanism for decanting single intensity carpets from larger files, which contain multiple carpets.
- The "Correlation Carpets to .tiff" button exports an image of correlated carpet which can be used for subsequent analysis in for example Matlab, python, Fiji/ImageJ.

19) 'Export to Fit' button exports current carpet to the fit interface.
- 'Export All Carpets to Fit' button exports all carpets in the data viewer to the fitting interface.
- 'Export All Carpets to CSV' button exports correlated data out of software.

20) 'Toggle Carpet Browser' this toggles between an independent widget containing all the loaded carpets and the inline browser. If you have many files open it can be easier to navigate your files using the independent widget window.

21) Within the correlated data window this displays the filename of the correlated files. The 'plot' checkbox shows which file is currently plotted.

22) The 'x' button will when pressed delete the corresponding correlated file.

Diffusion coefficient fitting interface

Once a data-file has been correlated it is desirable to calculate the coefficients of diffusion for that function. With FoCuS-scan, this can be performed using the included fitting software which applies Levenberg-Marquardt optimization to find the optimal parameters for a given model. The FoCuS-scan fitting functions can be accessed by clicking the ‘FCS Function Fitting' tab at the top of the software window. The following list describes the functionality of the main areas labeled below:

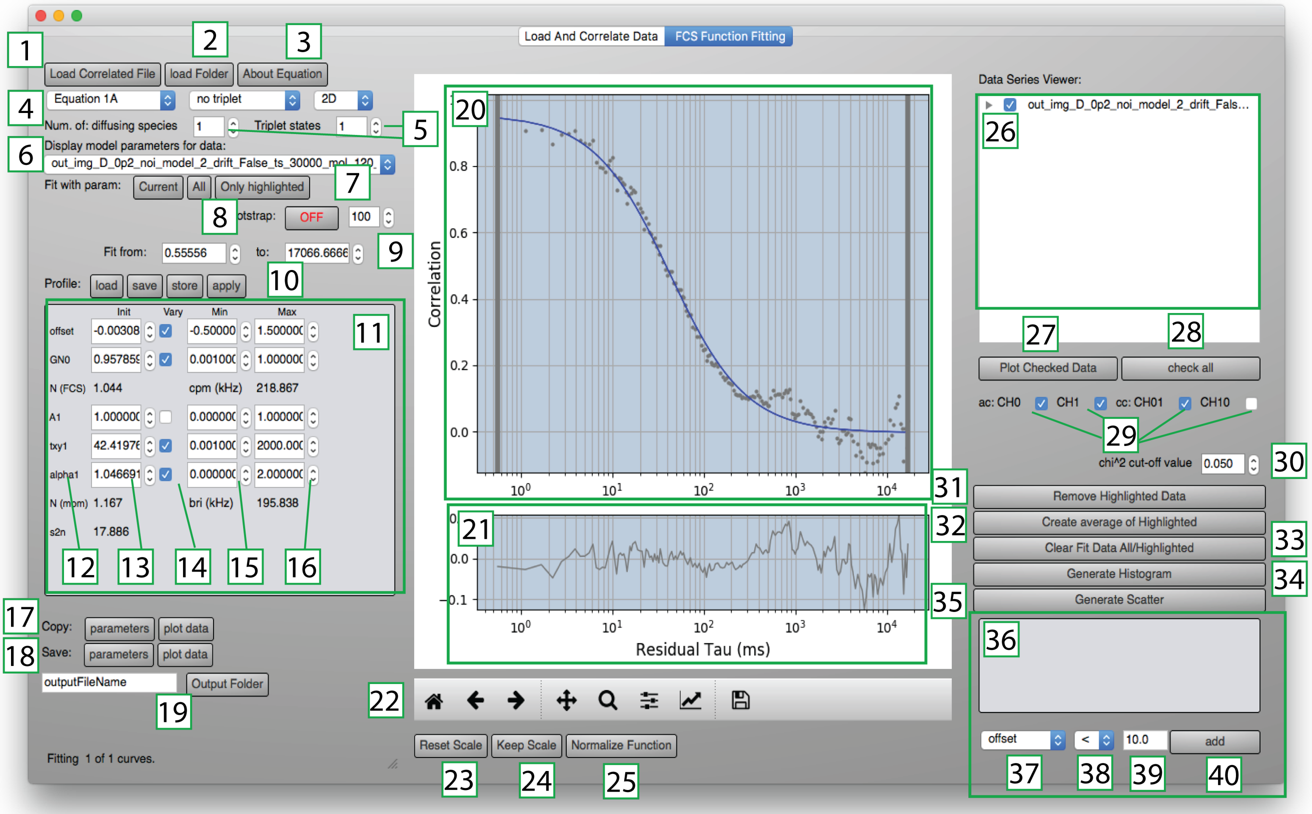

File import and fitting configuration.

1) Previously correlated data files, which have been saved, can be reloaded into the FoCuS-scan software using the 'Load Correlated File' button. Correlated files which have exported in this session with the ‘Export to Fit’ button will appear in the list on the right-hand side (26).

2) The ‘Load Folder’ button will load all the correlated data-files in an input folder into the fitting software.

3) The 'About Equation' button opens a text box which gives details relating to the equation used in the fitting software. Similar details are given in this document.

The drop-down lists, below the first row of buttons, provide access to the different fit equation options described in the next section of this guide (Diffusion coefficient calculation).

4) The first drop-down box with Equation 1A as default refers to the diffusion equations described in the next section (Diffusion coefficient calculation) and the way in which the axial diffusion component is handled when fitting a 3D diffusion equation. The second drop-down box, describes which of the two triplet state equations to use, either 'no triplet' or 'Triplet Eq. 2A' or 'Triplet Eq. 2B'. The final dropdown box sets whether 2D or 3D diffusion is used.

5) Once the equations have been chosen the number of diffusing species and triplet states can be selected in the next row of interface controls, 1-3 in both cases.

6) Every correlated pixel has fit parameters associated with it. To view the parameters of any of the correlated data, select it from the drop-down list 'Display model parameters for data'. When this data-file is selected in this list its parameters will appear below.

7) When clicked, the button marked 'Current' fits a curve to the selected data-file using the parameters depicted in (11). Whereas, the button marked 'All' will fit all of the correlation functions loaded in the data-series box (26) with the parameters currently shown in (11). The button marked 'Only highlighted' will fit those data-files that have been highlighted in the data-series box (26) with the displayed parameters.

8) The bootstrap option is by default off. If you click the ‘OFF’ button so that it turns ‘ON’ then for every curve a number of bootstrap samples of the data will be fit in parallel when one of the fitting buttons (7) is pressed. When ‘ON” the number of bootstraps is equal to the displayed counter (default: 100). Bootstrapping can help with generating statistics for curve fitting as the output standard deviation values represent error generated from bootstraps rather than the conventional fitting error.

9) The 'Fit from' and 'to' fields refer to time range which will be fit and is linked to the grey bar limits which are shown in the plot area (20).

10) For complicated experiments, where multiple fit parameters are changed from the defaults, it can be useful to save the fit profile to make it straight-forward to repeat the experiment. During a single session this can be done through using the ‘store’ button to save the currently used settings, and the ‘apply’ button to apply them. If the settings will be used for subsequent experiments it can be useful to write them onto the host computer, do this by using the ‘save’ button and then later loading them through using the ‘load’ button.

11) This section contains the parameters that are fit as well as the controls that control the fitting. The parameters that are visible are linked to the equation options that have been selected using the menus in (4-6).

12) The left-hand column specifies the variable name of this fit parameter. For full details see the next section (Diffusion coefficient calculation).

13) During the fitting each variable will be initiated at the value specified in the ‘Init’ column.

- During fitting those parameters that have the 'vary' check box (14) checked will be estimated by the fit algorithm within the ranges specified by the 'min' (15) and 'max' (16) boxes which are specified for each parameter.

The parameters and raw plot data for the data-series can be exported to the clipboard (17) or saved (18) using these buttons. If the Save buttons are clicked the data will be exported with the file prefix specified in (18) as well as to the specified ‘Output Folder’. If data is specifically highlighted in the data-series box (26) it alone will be copied and not all the data in the data series.

20) This is the main plot window. The data-files which has been checked in the data-series box '9' will appear here along with the corresponding fit function if these particular data-files have been fitted and their parameters estimated. By clicking and dragging the grey bars it is possible to restrict or increase the region being fit.

21) This plot window displays the residuals calculated from the fitting procedure for each of the plotted data-files.

22) Below the plot window is a Navigation Toolbar. The tools on the toolbar allow manipulation of the plotted correlation function and residuals. A) House: will return settings to default, click if you want to undo any changes you make to the visualisation. B and C) Arrows, will redo and undo changes in sequence. D) Multi-directional Arrows, when active, can be used to click and drag the visualisations. D) Zoom, when active allows the user to zoom in and out of the visualisations. E) Margins, allows the user to change the margins and padding for the visualisations (useful for creating figures). F) Disk, allows the user to save out an image of the correlation plots. G) Tick/Chart, Allows the user to format the visualisation of the plots and to specify ranges.

23) The 'Reset Scale' button resets the image to the initial image zoom.

24) The 'Keep Scale' button is very important for keeping the view static when fitting. Make sure the 'Keep Scale' button is highlighted to stop the plot window from changing zoom when replotting.

25) The ‘Normalize Function’ button will normalize the visualization of each curve to the same amplitude. Useful for visually comparing the transit time of the curves.

26) The Data-series box contains all the data-files which can be plotted and fit using the FoCuS-scan fitting software. The data-files, each representing a pixel are grouped by input file (i.e. by carpet). To plot a data-file correlation function, check the box associated with each name in the list, followed by clicking the 'Plot Checked Data’ button (27). Checked files will be plotted. Highlighting is different to checking. Highlighting files will affect the processing of the data-files whereas checking only affects the plotting.
Tip: To view the parameters of a correlated data file double-click the data-file name as listed here.
Tip: There are certain actions that can be performed on highlighted data. To highlight data, left-click and select multiple data-file names.

Tip: To highlight all the data-files press Ctrl-A (Windows) or Cmd-A (mac) keyboard.
-To check all data click the 'check all' button (28) located below the list.
-To uncheck all checked data click the 'check none' button located below the list.

29) This set of check boxes represent the different correlation channels to be included or hidden in the data-series box, enabling them or not for plotting or fitting. CH0 refers to 1st channel, CH1 refers to the 2nd channel, CH01 refers to the cross-correlation with respect to the 1st channel to the 2nd and the CH10 represents the alternate cross-correlation function.

30) Upon fitting the data-files are colour coded to denote whether the fit was good or not. The chi^2 defining a good or bad fit can depend on the data and model being used. By changing the value in this box the criteria for a good or bad fit can be altered. The following buttons perform actions on highlighted data-files in the data-series box (26).

31) The 'Remove Highlighted Data' button will delete highlighted correlated data-files from FoCuS-scan.

32) The ‘Create average of Highlighted’ button will generate a new average data-file from all of the highlighted data-files.

33) The 'Clear Fit Data All/Highlighted' will clear all parameters which have been estimated during fitting or will clear those of only the highlighted data-files should any be highlighted.

34) The 'Generate Histogram' button and the 'Generate Scatter' (35) buttons generate visualisations which can be used to assess the distribution of fit parameters across all the data-files.

36) The box below allows filters to be applied to the data being plotted allowing the user to differentially visualize their data. The associated filter buttons allow specific filters to be setup and applied in different ways.

37) The first drop-down box specifies the fit parameter to be used as the basis of the filter.

38) Specifies the direction of the filter, i.e parameters values to be filtered are ‘<’ less than or ‘>’ greater than the specified value in (39).

40) The ‘add’ button creates the filter.

Tip: Once a filter is setup. The effect of the filter can be controlled using the toggleable 'apply/show/off' button. If the text 'Apply' is shown it means that the data from the viewer is excluded by the filter, 'off', turns the filtering off. 'show' includes the filtered data, but makes it translucent, so that it can be visualized against the rest of the data.

Diffusion coefficient calculation

FoCuS-scan has a number of options for configuring the equation that is used to fit the calculated correlation function. Within FoCuS-scan, it is possible to fit an equation with either 2D or 3D diffusion equations with between one to three diffusion components. There is also the option to include, one to three triplet states in the equation also (although 'no triplet 'is the default). Triplet state equations are used to model the cases when the fluorophores under investigation have dark-states that can be induced by the imaging regime. If present it is normal to correct for triplet states in the diffusion equation, due to the impact they have on the overall correlation curve. The overall equation for analyzing the correlation function is

$$G_N(\tau) = O_f + G_N(0)[G_D(\tau).G_T(\tau)] \quad (9)$$

where τ represents time, O_f represents the offset (normally zero), G_N (0) is the amplitude of the correlation function, G_D is the diffusing component and G_T is the optional triplet state. For 2D diffusion (e.g. in a bi-layer) G_{2D} is used (in place of G_D)

$$G_{2D}(\tau) = \sum_{k=1}^{D_s} A_k((1 + (\tau_c/\tau_{xyk})^{\alpha^k})^{-1}) \quad (10)$$

and between one and three diffusing species are included using the D_S parameter. For the case D_S = 1, A_1 = 1, else $\sum^{D_s} A_k = 1$ and this parameter k=1 serves to establish how the amplitude is composed of the different diffusing species. τxy_k is the lateral diffusion rate coefficient and represents the time taken for the diffusing species to move laterally through the illumination area. α is the anomalous factor which is important for compensating for when the diffusion kinetics are non-idea. For ideal diffusion, the anomalous factor = 1.0 whereas if, for example, Continuous Wave STED depletion is used this factor can drop towards 0.0 unless time-gating is employed. A similar factor is not included for the z-dimension at this time as anomalous diffusion from conventional STED illumination is only potent in the z-axis. For studying cases of 3D diffusion, FoCuS-scan has two different equations at its disposal:

$$G_{3D}(\tau) = \sum_{k=1}^{D_s} A_k((1 + (\tau/\tau_{xyk})^{\alpha^k})^{-1})(1 + (\tau/\tau_{zk}))^{-1/2} \quad (1A)$$

and

$$G_{3D}(\tau) = \sum_{k=1}^{D_s} A_k((1 + (\tau/\tau_{xyk})^{\alpha^k})^{-1})(1 + (\tau/(AR_k^2.\tau_{xyk})))^{-1/2}$$

(1B)

τ_{zk} is the transit time in the axial dimension of each species. The reason you might use the second G_{3D} equation is that in ideal cases τ_z is related to τ_{xy} through some constant factor (AR) and so it is desirable to just find AR rather than trying to learn τ_z that is dependent on τ. The anomalous factor is not applied to the calculation of τ_z as often the dominant dimension of diffusion is in the lateral dimension and so the contribution of the anomalous factor in the axial-dimension is considered to be trivial. Finally, FoCuS-scan offers two triplet equations. insert more information relating to these.

$$G_T(\tau) = 1 + \sum_{j=1}^{Ts} B_j.exp(-\tau/\tau_{Tj}) \ , \ B_j = T_j/(1 - T_j)$$

(2A)

$$G_T(\tau) = 1 + \sum_{j=1}^{Ts} T_j + \sum_{j=1}^{Ts} T_j.exp(-\tau/\tau_{Tj})$$

(2B)

The fitting itself in FoCuS-scan is performed using a Levenberg-Marquardt algorithm from the lmfit python library (https://lmfit.github.io/lmfit-py/). In addition to the parameters derived directly from fitting the diffusion equations mentioned above there are also some other parameters which are indirectly calculated by which are equally useful.

With the above models it is possible to calculate the diffusion coefficient with knowledge of the detection beam radius and the measured transit time using: $D_k = \omega_{xy}^2/4.\tau_{xyk}$, where D_k is the diffusion coefficient ($\mu m^2.s^{-1}$) of species k and ω_{xy} is the lateral beam radius (μm). The lateral beam radius can be calculated from the beam FWHM with: $\omega_{xy} = FWHM/\sqrt{2.In(2)}$. Furthermore, the diffusion coefficient can also be calculated from the axial transit time measurement with $D_k = \omega_z^2/4.\tau_{zk}$ where $\omega_z = FWHM_z/\sqrt{2.In(2)}$ and $FWHM_z$ is the FWHM in the axial direction.

To assist in the calculation and interpretation of cross-correlation data FoCuS-scan also offers coincidence analysis through:

$$Coincidence = \frac{\sum IH_0.IH_1}{\sum IH_0 . \sum_t IH_1}$$

(3)

, where IH_0 and IH_1 are intensity histograms calculated from the first and second raw input intensity data in a given spatial location. This equation is a form of brightness analysis that correlates the coincidence of brightness within the acquired channels.

Amplitude ratio measurements can be used to assess the relative abundance of species in an experiment:

$$ACAC_{01} = G_N^{AC0}(0)/G_N^{AC1}(0) \quad (4A)$$

and

$$ACCC_{01} = G_N^{AC0}(0)/G_N^{CC01}(0)\ , \quad (4B)$$

where $ACAC_{01}$ is the amplitude ratio of the first channel with respect to the second and $G_N^{AC0}(0)$ is the amplitude measurement from the first autocorrelation channel and $G_N^{AC1}(0)$ is the same measurement from the second channel. $ACCC_{01}$ is similar but represents the amplitude ratio of the first channel to the amplitude calculated from cross-correlation function amplitude $G_N^{CC01}(0)$.

Conclusion

FoCuS-scan allows bulk correlation and fitting of raw scanning data so that it can be analysed as efficiently as possible using the state-of-the-art techniques. This document has outlined the interface of the software and provided technical details as to the specification of the algorithms used for correlation and fitting.
